# Selection-Independent Generation of Gene Knockout Mouse Embryonic Stem Cells Using Zinc-Finger Nucleases

**DOI:** 10.1371/journal.pone.0028911

**Published:** 2011-12-14

**Authors:** Anna Osiak, Frank Radecke, Eva Guhl, Sarah Radecke, Nadine Dannemann, Fabienne Lütge, Silke Glage, Cornelia Rudolph, Tobias Cantz, Klaus Schwarz, Regine Heilbronn, Toni Cathomen

**Affiliations:** 1 Institute of Experimental Hematology, Hannover Medical School, Hannover, Germany; 2 Institute of Virology (CBF), Charité Medical School, Berlin, Germany; 3 Institute for Transfusion Medicine, University of Ulm, Ulm, Germany; 4 Institute for Clinical Transfusion Medicine and Immunogenetics, German Red Cross Blood Service Baden-Württemberg – Hessen, Ulm, Germany; 5 Institute for Laboratory Animal Science, Hannover Medical School, Hannover, Germany; 6 JRG Genetic and Epigenetic Integrity, REBIRTH Cluster of Excellence, Hannover Medical School, Hannover, Germany; 7 JRG Stem Cell Biology, REBIRTH Cluster of Excellence, Hannover Medical School, Hannover, Germany; University of Southern California, United States of America

## Abstract

Gene knockout in murine embryonic stem cells (ESCs) has been an invaluable tool to study gene function *in vitro* or to generate animal models with altered phenotypes. Gene targeting using standard techniques, however, is rather inefficient and typically does not exceed frequencies of 10^−6^. In consequence, the usage of complex positive/negative selection strategies to isolate targeted clones has been necessary. Here, we present a rapid single-step approach to generate a gene knockout in mouse ESCs using engineered zinc-finger nucleases (ZFNs). Upon transient expression of ZFNs, the target gene is cleaved by the designer nucleases and then repaired by non-homologous end-joining, an error-prone DNA repair process that introduces insertions/deletions at the break site and therefore leads to functional null mutations. To explore and quantify the potential of ZFNs to generate a gene knockout in pluripotent stem cells, we generated a mouse ESC line containing an X-chromosomally integrated *EGFP* marker gene. Applying optimized conditions, the *EGFP* locus was disrupted in up to 8% of ESCs after transfection of the ZFN expression vectors, thus obviating the need of selection markers to identify targeted cells, which may impede or complicate downstream applications. Both activity and ZFN-associated cytotoxicity was dependent on vector dose and the architecture of the nuclease domain. Importantly, teratoma formation assays of selected ESC clones confirmed that ZFN-treated ESCs maintained pluripotency. In conclusion, the described ZFN-based approach represents a fast strategy for generating gene knockouts in ESCs in a selection-independent fashion that should be easily transferrable to other pluripotent stem cells.

## Introduction

Since its introduction some 30 years ago, targeted gene editing in embryonic stem cells (ESCs) has dramatically changed biomedical research. Although targeted genome engineering was mainly restricted to murine ESCs for more than two decades, these cells have served as excellent model systems to study gene function *in vitro* or to generate knockout and knock-in mouse models [1]. Because gene targeting in mouse ESCs using standard techniques is rather inefficient and typically does not exceed frequencies of ∼10^−6^ [2], the application of complex positive/negative selection strategies to isolate targeted clones has been inevitable. With the availability of induced pluripotent stem cells (iPSCs) [3] and improved gene targeting technologies, targeted genome engineering could be transferred to other organisms, including human cells. For instance, gene targeting in human ESCs or iPSCs has been successfully accomplished with vectors based on integrase-deficient lentivirus [4], adeno-associated virus [5], [6], adenovirus [7], baculovirus [8], and non-viral systems, such as bacterial artificial chromosomes [9]. Furthermore, it has been established that the frequency of gene targeting at a marker gene in mouse ESCs could be significantly augmented by creating a targeted DNA double-strand break with the natural homing endonuclease I-SceI [10], a concept that could be expanded using custom-made zinc-finger nucleases (ZFNs) to correct a mutated *EGFP* locus [11]. Recent reports demonstrate that ZFNs also allowed for the generation of human iPSC lines that either emulate or correct a disease genotype/phenotype [12], [13], [14]. However, even though many of these novel approaches proved to increase the gene targeting frequency in pluripotent stem cells considerably, all of them – even with designer nucleases – were still dependent on either positive selection markers to enrich for targeted cells or on screening of large numbers of clones.

ZFNs are the most successful class of designer nucleases up till now, with one ZFN pair in clinical trials (*e.g.* NCT01252641). A ZFN is a functional heterodimer [15], and each subunit consists of a non-specific nuclease domain derived from the FokI endonuclease and a specific DNA-binding domain composed of an engineered zinc-finger array that tethers the enzyme to a preselected chromosomal site [16]. Upon dimerization of two ZFN monomers at the target site, the ZFN pair specifically cleaves the DNA. The resulting double-strand break triggers the cellular DNA damage response, which can be harnessed for gene targeting by homologous recombination (HR) or gene knockout by non-homologous end-joining (NHEJ) [17], [18]. Recent progress in the architectural design of ZFNs have led to both an increase in nuclease activity and a substantial decrease in nuclease-associated toxicity [19]. The main improvements include perfected platforms to generate the DNA-binding domains [18], [20], [21], [22], remodeling of the nuclease dimer interface to prevent homodimerization of two identical ZFN monomers [23], [24], [25], [26], [27], and customized linkers that connect these two main domains [28], [29]. As recently demonstrated for a well-characterized ZFN that was designed to target the human *CCR5* locus, activity at the target site is at least 3 times more likely than at all off-target sites combined [30], [31].

In this study, we aimed at developing a simple and efficient method to generate pluripotent knockout cells without the use of selection systems. While the ZFN technology has been employed to generate knockouts in a few model organisms and primary cell types (summarized in [32]), efficient selection-independent approaches in pluripotent cells have not been described thus far. In a proof-of-concept approach, we used ZFNs to disrupt the open reading frame of an X-chromosomally located *EGFP* gene. We demonstrate that a knockout could be achieved in up to 8% of transfected mouse ESCs and that ZFN-treated cells preserved both normal chromosomal numbers and pluripotency.

## Results

### ZFN activity and toxicity

**Figure 1:**
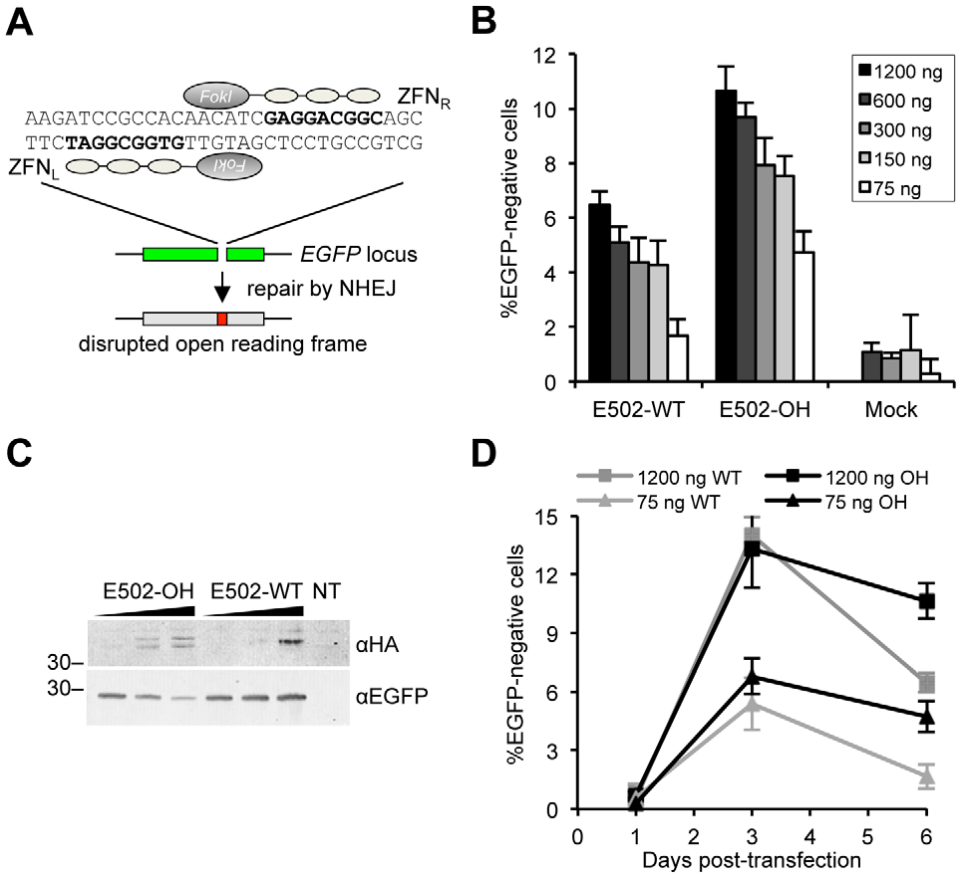
Gene knockout in U2OS.693 cells. (**A**) Schematic of ZFN-mediated knockout. A ZFN pair (ZFN_R_ and ZFN_L_) designed to target position 502 in the *EGFP* gene (E502) creates a DNA double-strand break that is sealed by the error-prone non-homologous end-joining (NHEJ) pathway and hence leads to disruption of the coding sequence. (**B**) Dose-dependent gene disruption in U2OS.693 cells. U2OS.693 cells that stably express a destabilized EGFP were transfected with increasing amounts of E502-specific ZFN expression vectors (75–1200 ng). The percentage of EGFP-negative cells was determined 6 days post-transfection by flow cytometry (n = 3; indicated is average and standard deviation). E502-WT, ZFN with wild-type FokI domain; E502-OH, ZFN with obligate heterodimeric FokI domain. (**C**) ZFN expression levels. After co-transfection of HEK293T cells with ZFN expression vectors and pEGFP, cell lysates were probed with antibodies against the HA tag and EGFP. Amount of transfected ZFN plasmids was 75 ng, 300 ng, and 1200 ng. NT, non-transfected cells. (**D**) Kinetics of *EGFP* knockout. The graph displays the percentage of EGFP-negative cells (see B) from day 1 to day 6 post-transfection for two vector amounts (n = 3; indicated is average and standard deviation). WT, E502-WT; OH, E502-OH.

For initial characterization, a human osteosarcoma-based cell line expressing a destabilized EGFP (U2OS.693) was used to assess the effects of vector dose and nuclease type on gene knockout activity and ZFN-associated toxicity. The two *EGFP*-specific ZFN pairs target position 502 in the *EGFP* open reading frame [33] (**Fig. 1A**) and harbor either a wild-type FokI nuclease domain (E502-WT) or an obligate heterodimeric (E502-OH) variant [23], [25]. Upon co-transfection of U2OS.693 cells with increasing amounts of ZFN expression plasmids (75–1200 ng/well) and an mCherry expression vector as an internal reference, a clear dose response was observed (**Fig. 1B**). At day 6 post-transfection, the percentage of EGFP-negative cells ranged from 1.7–6.5% for E502-WT and 4.7–10.7% for E502-OH. A transfection with a control vector (Mock) served to determine the background of EGFP-negative cells in the culture. Analysis of ZFN expression confirmed comparable expression levels of E502-WT and E502-OH (**Fig. 1C**). Note, as opposed to E502-WT the two subunits of E502-OH migrate differently in SDS-PAGE [23], [25]. Examination of the kinetics of ZFN-mediated *EGFP* knockout revealed that although the percentage of gene knockout was similar at day 3 with ∼14% of EGFP-negative cells, this fraction declined by >50% for E502-WT and ∼20% for E502-OH treated cells until day 6, respectively (**Fig. 1D**), likely due to the effect of off-target events in these cells. Together these data show that the efficiency of ZFN-mediated gene disruption increased with the ZFN vector dose and decreased with nuclease-associated toxicity, especially with the WT variant. Although the cytotoxicity was considerably reduced when using obligate heterodimeric nuclease domains, the used ZFN pair may still exhibit a considerable degree of unwanted cytotoxic effects.

### ZFN mediated knockout in mouse embryonic stem cells

**Figure 2:**
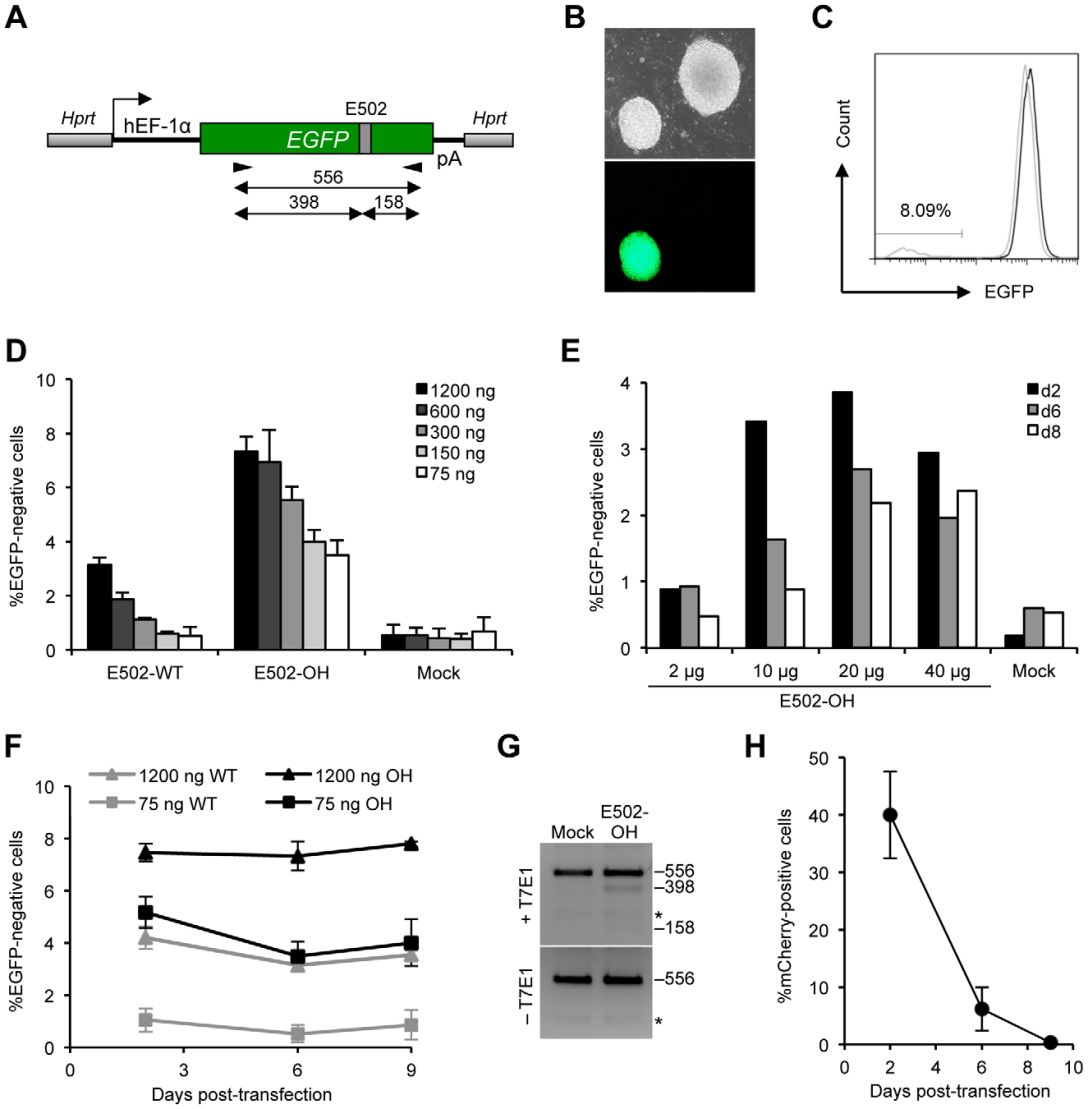
Gene knockout in mouse embryonic stem cells. (**A**) Schematic of *EGFP* locus in BK4-G3.16 cells. The mouse ESC line BK4-G3.16 contains an *EGFP* gene stably integrated 5′ of the endogenous *Hprt* locus. E502, ZFN target site; hEF-1α, human elongation factor 1α promoter; pA, poly(A). The primer binding sites and the length (in base pairs) of the PCR amplicon are indicated. (**B, C**) Assessment of EGFP expression in BK4-G3.16 cells. Marker gene expression was determined by fluorescence microscopy (B) or flow cytometry (C) and was used to identify knockout clones (B) or to quantify gene disruption frequencies (C). (**D, E**) Dose-dependent gene disruption in mouse ESCs. BK4-G3.16 cells were lipofected (D) or electroporated (E) with increasing amounts of E502-specific ZFN expression vectors and an mCherry plasmid as an internal reference. The percentage of EGFP-negative cells was determined by flow cytometry at day 6 (D; n = 3; indicated is average and standard deviation) or at days 2, 6, and 8 (E; n = 1) post-transfection. (**F**) Kinetics of *EGFP* knockout. The graph displays the percentage of EGFP-negative cells (see D) from day 2 to day 9 post-transfection for two vector amounts (n = 3; indicated is average and standard deviation). WT, E502-WT; OH, E502-OH. (**G**) ZFN-induced mutations at E502 site. After lipofection of BK4-G3.16 with E502-OH expression vector, a genomic PCR fragment encompassing target site E502 was digested with the mismatch-sensitive T7 endonuclease I (T7E1). The expected lengths (in base pairs) of the T7E1 digestion products are shown on the right. Asterisks indicate an unspecific DNA fragment. Transfection with a vector control (Mock) was used as a control. (**H**) Expression kinetics. The graph displays the percentage of mCherry-positive BK4-G3.16 cells (see D) from day 2 to day 9 post-transfection.

To assess the feasibility of disrupting a gene in pluripotent stem cells, the hemizygous mouse ESC line BK4-G3.16 carrying a single copy marker was generated by targeting integration of an *EGFP* gene under control of the hEF-1α promoter into the X-chromosomal *Hprt* locus (**Fig. 2A**). EGFP expression in the BK4-G3.16 ESC line is high and allows for easy distinction of EGFP-negative from positive cells by fluorescent microscopy (**Fig. 2B**) or flow cytometry (**Fig. 2C**).

To knock out marker gene expression, 3×10^5^ BK4-G3.16 ESCs were lipofected with increasing amounts of ZFN expression plasmids (75–1200 ng/well) and an mCherry expression vector as an internal reference for ZFN production. Flow cytometric analysis at day 6 post-transfection revealed a clear dose response, with 0.3–3.2% of EGFP-negative cells when using ZFN variant E502-WT and 3.3–8% of gene disruption with E502-OH (**Fig. 2D**). Because electroporation has been the method of choice to transfer nucleic acids into mouse ESCs, we compared the efficiency of this method with lipofection. To this end, 1×10^7^ BK4-G3.16 cells were co-electroporated with 2–40 µg of E502-OH expression vector and the mCherry plasmid. The percentage of EGFP-negative cells was determined between day 2 and 8 post-transfection (**Fig. 2E**). Maximal gene disruption activity was achieved with 20 µg of E502-OH plasmid, leading to ∼4% of EGFP-negative cells at day 2 after electroporation. The percentage of EGFP-negative cells slightly decreased during the observation period of 8 days. Similar results were obtained in a second mouse ESC line that harbors an *EGFP* gene integrated into the *Rad54* locus (data not shown). The lower gene knockout frequency in electroporated ESCs can partially be explained by the lower overall transfection efficiency, with ∼40% mCherry-positive cells after lipofection and ∼30% upon electroporation (data not shown), respectively.

An evaluation of the kinetics of ZFN-mediated gene disruption in lipofected ESCs demonstrated that the percentage of EGFP-negative cells remained stable over the time frame of 9 days (**Fig. 2F**). Unlike U2OS.693 cells, however, E502-WT and E502-OH treated ESCs did not show a similar gene disruption frequency at early time points. This implies that ESCs may be more sensitive to ZFN off-target activity and cells exposed to high E502-WT levels were probably lost early after transfection. To confirm gene disruption on the genome level, total DNA was extracted three days after lipofection and the *EGFP* locus subjected to analysis with the mismatch-sensitive T7 endonuclease I (T7E1). The fraction of mutated alleles after exposure of cells to ZFN E502-OH (**Fig. 2G**) was in good agreement with the gene disruption frequency determined by flow cytometry (**Fig. 2D**). Finally, analysis of the mCherry expression levels, as a surrogate marker for ZFN expression, verified that transgene expression in ESCs is transient and rapidly lost in these fast dividing cells (**Fig. 2H**).

In conclusion, similar to the results in the U2OS.693 cells, our data from the ESCs show that the efficiency of ZFN-mediated gene disruption was increased when elevated amounts of ZFNs were expressed, while they were reduced when the more cytotoxic E502-WT variants were employed. However, the fraction of knockout cells after lipofection of the ZFN expression plasmids remained stable, suggesting that cell viability stabilized after the initial exposure to the nuclease. This observation is in accordance with the rapid dilution of expression vectors in fast dividing ESCs.

### Characterization of ZFN treated embryonic stem cells

**Figure 3:**
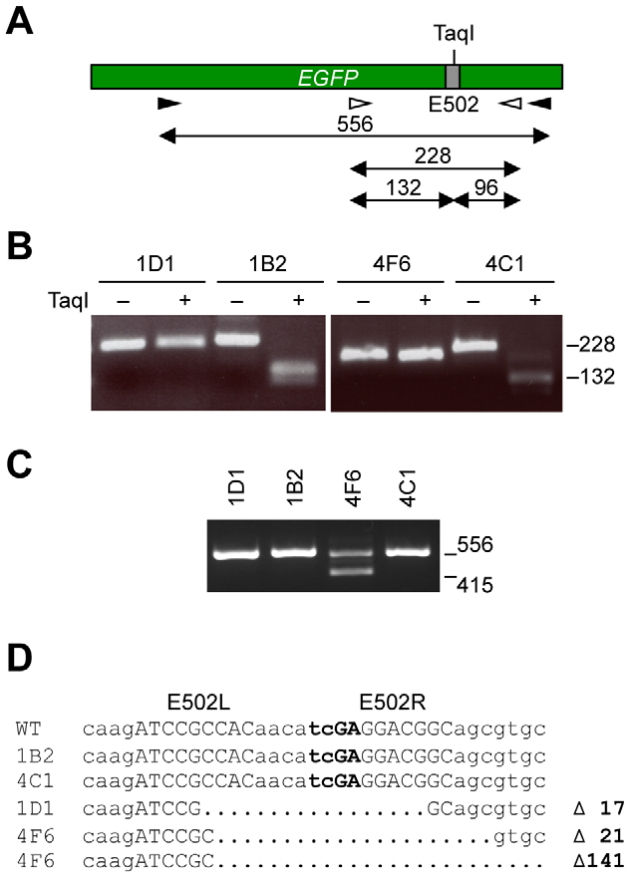
Molecular characterization of individual ESC clones. (**A**) Schematic of *EGFP* locus in BK4-G3.16 cells. The primer binding sites and the lengths (in base pairs) of the generated PCR amplicons are indicated. (**B**) Genotyping. The genomic PCR amplicon encompassing target site E502 was digested with TaqI. The positions of the TaqI-resistant DNA fragment (228 bp) and a cleavage product (132 bp) are indicated. (**C, D**) Sequence of disrupted *EGFP* alleles. A genomic PCR expected to produce a 556-bp amplicon was evaluated by agarose gel electrophoresis (C) and the isolated DNA fragments sequenced (D). The positions of the expected (556 bp) and the additional DNA fragment (415 bp) are indicated in (C), the sequences of all alleles are shown in (D). The ZFN target site (subsites E502L and E502R) is highlighted in capital letters, the TaqI site in bold letters.

To scrutinize the impact of ZFN expression on electroporated pluripotent mouse cells, two single EGFP-negative ESC clones, termed 1D1 and 4F6, and two EGFP-positive clones, termed 1B2 and 4C1, were subjected to further analyses. To genotype these clones, two genomic PCR reactions producing either 228 or 556-bp amplicons, respectively, that include the E502 target site were performed (**Fig. 3A**). The 228-bp fragment contains a unique TaqI site that overlaps with the ZFN recognition sequence, and error-prone repair of the ZFN-induced DNA double-strand break at E502 can lead to disruption of the restriction site. Indeed, the genomic PCR amplicons generated from the knockout clones were resistant to cleavage with TaqI endonuclease (**Fig. 3B**). When the external primers were used for genomic PCR, a 415-bp fragment was produced in addition to the expected 556-bp amplicon from DNA extracted from clone 4F6 (**Fig. 3C**). Sequencing of these PCR products confirmed that the EGFP-positive clones 1B2 and 4C1 retained the wild-type sequence (**Fig. 3D**), while the *EGFP* gene in clone 1D1 harbored a 17-bp deletion. The two *EGFP* alleles in 4F6 cells contained deletions of 21 and 141 bp, respectively. These sequencing results for clones 1D1 and 4F6 are consistent with both resistance of the PCR amplicon to TaqI digestion (Fig. 3B) and the smaller size of the PCR fragments (Fig. 3B,C).

**Figure 4:**
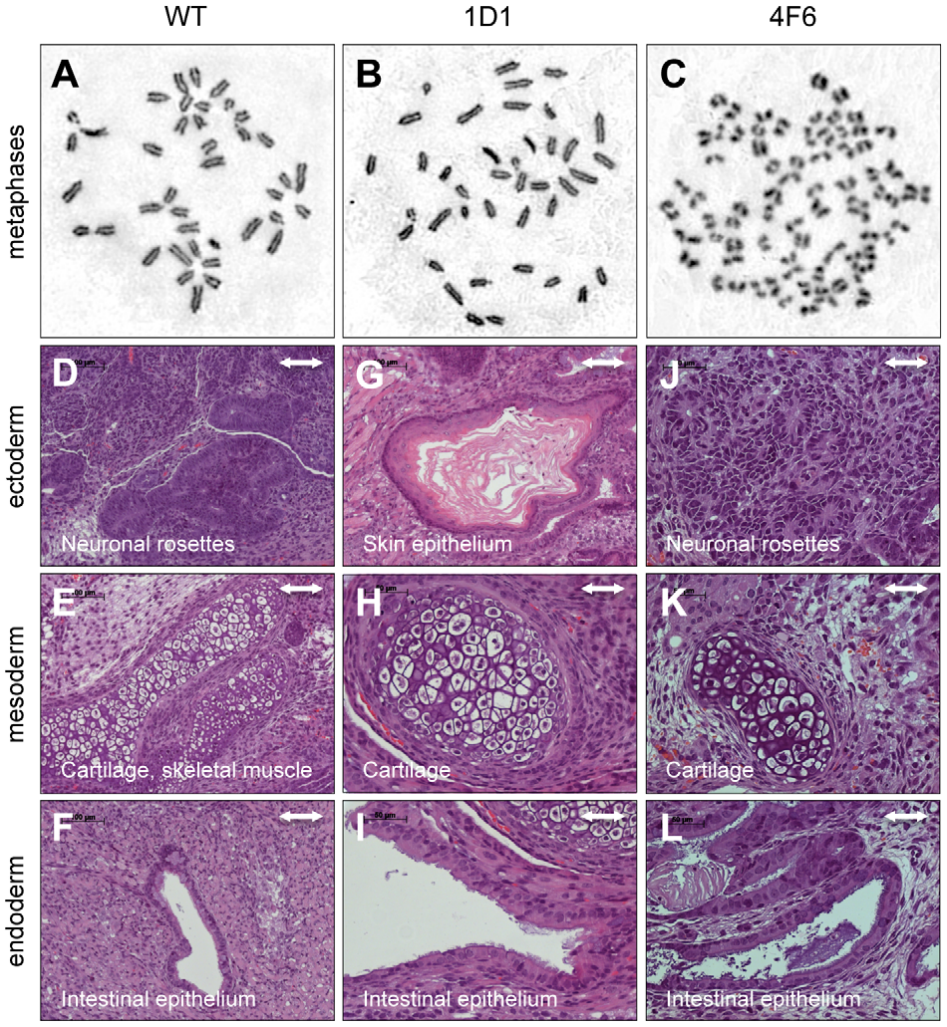
Assessment of metaphase chromosomes and pluripotency. (**A–C**) Chromosome analysis. Metaphase spreads of BK4-G3.16 cells were stained with Giemsa. A representative picture of ∼20 analyzed metaphase spreads per clone is shown. (**D–L**) Assessment of pluripotency. ZFN-treated BK4-G3.16 cells were injected subcutaneously into immunodeficient mice and teratomas removed after 4–8 weeks. Histological analysis using hematoxylin/eosin staining revealed tissues derived from ectoderm (D, G, J), mesoderm (E, H, K) and endoderm (F, I, L). Scale bars = 100 µm for (D–G) and 50 µm for (H–L).

Because the presence of two *EGFP* alleles in the 4F6 cells was rather unexpected, the number of chromosomes per cell was assessed from ∼20 metaphase spreads for each clone after Giemsa staining. While untreated cells and clone 1D1 cells appeared normal with 40 chromosomes each (**Fig. 4A, B**), 4F6 cells contained between 53 and 90 chromosomes (**Fig. 4C**). The increase in chromosome number in 4F6 cells offered an explanation for the presence of two *EGFP* alleles. It is not clear at this point, however, whether the variable number of chromosomes in 4F6 cells was triggered by ZFN expression or was a reflection of genomic instability caused by long-term culturing of the cells [**34**].

Finally, we assessed whether the ZFN treated cells had preserved pluripotency. To this end, ZFN-treated ESC clones 1D1 and 4F6 were injected subcutaneously into immunodeficient mice. Teratomas were removed after 4–8 weeks and analyzed histologically. Both 1D1 and 4F6 knockout ESCs induced the formation of teratomas that contained all three germ layers. However, as opposed to teratomas derived from wild-type ESCs (**Fig. 4D–F**) or clone 1D1 cells (**Fig. 4G–I**), the tumors from 4F6 cells developed with a delay of ∼4 weeks. The ability of 4F6 cells to differentiate into all three germ layers (**Fig. 4J–L**) suggests either the presence of some few normal diploid cells that were not detected in the limited number of analyzed metaphase spreads or that the polyploid state of 4F6 cells did not abrogate the teratoma formation capability. Together, these data show that ZFNs can be used to efficiently disrupt a gene in mouse ESCs and that individual clones with normal chromosome numbers and preserved pluripotent state can be identified.

## Discussion

Gene knockout is a powerful tool for either assessing gene function and/or permanently modifying the phenotype of a cell or an entire organism. In selection-free approaches, the ZFN technology has been successfully used to generate NHEJ-based knockouts in various model organisms by injecting ZFN-encoding mRNA in the zygote or early embryo (summarized in [32]), including fruit fly [35], zebrafish [36], [37], rat [38], [39] and mouse [40]. Moreover, the generation of knockout cells has been reported but application of the ZFN technology has remained restricted to a few cell types, such as transformed CHO cells [41] or human T cells [42]. Here, we demonstrated that transient expression of engineered ZFNs is sufficient to generate a gene knockout in pluripotent stem cells and that chosen mouse ESC clones preserved both normal chromosome numbers and pluripotency.

Under optimized conditions, almost 1 out of 10 mouse ESCs contained a ZFN-induced mutation in *EGFP*. The highly specific and efficient mutagenic potential of ZFNs thus presents this simple one-step approach as a valid alternative to conventional knockout strategies for researches interested in the generation of knockout animals or knockout ESC lines. The simplicity of the process is based on two facts: First, in mammalian cells the NHEJ pathway is preferred over HR to seal DNA double-stand breaks [43]. Second, the error-prone nature of the NHEJ repair process frequently results in insertions/deletions at the ZFN-induced DNA break [44], which in turn is sufficient to disrupt gene expression. Our sequencing results are in good agreement with earlier reports and confirm that ZFN-induced mutations lead to frameshifts and/or large deletions that abrogate function of the protein of interest [41], [42]. As a consequence, and as opposed to the conventional HR-based gene knockout approaches in mouse ESCs, the ZFN-based strategy abrogates the need to design and generate complex targeting vectors for HR. This greatly simplifies the knockout protocol and eliminates the usage of selection markers that may impede or complicate downstream applications.

The construction of the necessary ZFNs to disrupt the locus of choice may have been a time consuming factor in the past, when complex selection strategies were necessary to produce ZFNs [22]. The availability of alternative platforms based on pre-assembled two-finger archives [45], [46] reduces the timeframe for generating functional ZFNs to a few weeks with little hands-on time. Moreover, alternative designer platforms based on transcription activator-like effector nucleases (TALENs) [47] will further reduce the production time to create a functional nuclease to about a week [48], [49], [50]. Interestingly, a first side-by-side comparison between ZFNs and TALENs suggest that the latter may be more specific and less cytotoxic [51].

The frequency of gene disruption in transformed human U2OS cells and pluripotent mouse ESCs was dependent on both the amount of ZFN expression plasmid transfected into the cells and the nuclease architecture. Likewise, ZFN-associated cytotoxicity correlated with ZFN dose and the nuclease variant employed. It has been shown previously that the use of obligate heterodimeric ZFN variants greatly reduced ZFN-associated toxicity in cultured cells [23], [24], [25], [26], [27] and in zebrafish embryos [52]. Moreover, it has been shown that obligate heterodimeric ZFN are more specific than their counterparts with a wild-type FokI nuclease domain [30]. Here we compared directly the activities and toxicities of ZFNs in an immortalized cell line and pluripotent stem cells. Although the knockout frequency of ZFNs with wild-type nuclease domain and obligate heterodimeric ZFNs may be similar at early time points, the percentage of EGFP-negative cells after treatment with E502-WT dropped substantially at later time points, suggesting massive toxicity. Our data hence emphasize the importance of employing obligate heterodimeric ZFN variants, even if ZFNs with optimized DNA-binding domains [22] are applied, as in this study. Although the use of obligate heterodimeric ZFNs reduces off-target cleavage, it cannot be fully prevented [30], [31]. However, the genome of individual ZFN-targeted ESC clones can be extensively characterized on a molecular level to identify clones without undesired mutations, if necessary. While this point might be a minor concern when generating knockout mice because the animals can be backcrossed to obtain a pure background, higher specificity of the designer nucleases that results in a “clean” undisturbed genome is important if the technology is to be used to knockout genes in human iPSCs for further use in therapeutic applications [53].

In conclusion, owing to their plasticity and potential to differentiate in all cell types, pluripotent stem cells represent valuable tools for investigating the early pathophysiology of genetic disorders and can serve as both cellular *in vitro* models for drug screening and cell therapeutics in regenerative medicine. As transient expression of the designer nucleases is sufficient to create the targeted DNA double-strand break, the ZFNs could also be applied in the form of mRNA [40]. Because of the high gene knockout efficiency, selectable markers, which may obstruct downstream applications, are not necessary to isolate targeted cells. The simplicity of the described technique will also permit the knockout of multiple genes, either sequentially or in parallel with autonomous ZFN pairs [25]. Furthermore, given that efficient transfection methods for human iPSCs have been established, the transfer of our findings to the human system should be straightforward. Future studies will be testing these concepts.

## Materials and Methods

### ZFN expression plasmids

The *EGFP*-specific ZFN expression vectors were generated by subcloning a ZFN pair targeting position 502 of the *EGFP* open reading frame (target site 5′-ATCCGCCACnnnnnnGAGGACGGC) [22] into pRK5 vectors [54] that contain either wild-type or the obligate heterodimeric KV/EA FokI variants [23], respectively. Maps and sequences of all plasmids are available upon request.

### Cell lines

U2OS.693 is a human osteosarcoma based cell line (ATCC® #HTB-96™) [51] that expresses a destabilized EGFP [55]. The polyclonal cell line was generated by lentiviral transduction followed by selection with 0.4 mg/ml geneticin [21]. Quantitative PCR established that cells contain between 3 and 7 copies of the provirus. U2OS.693 cells were cultured in Dulbecco's modified Eagle's medium (DMEM) supplemented with 10% fetal bovine serum (FBS) and penicillin/streptomycin (Invitrogen). The murine ESC line BK4-G3.16 is a derivative of the BK4 line (kindly provided by O. Smithies, University of North Carolina, Chapel Hill) which constitutes a subclone of the E14TG2a line (mouse strain background: 129/Ola) [56]. The construction of the targeting vector pMP8EGFP, a derivative of pMP8-SKB (kindly provided by O. Smithies), has previously been described [57]. The scaffold of the vector enables targeting of the murine X-chromosomal 5′-deleted *Hprt* locus. Homology-directed integration functionally reconstitutes this locus, allowing selection of targeted cell clones in the presence of hypoxanthine, aminopterin, and thymidine (HAT). At the same time, the *EGFP* reporter gene under control of the human elongation factor 1α (hEF-1α) promoter was introduced 5′ of the *Hprt* locus in the same orientation. After targeting and expansion, counting the chromosome number for clone BK4-G3.16 revealed 7 out of a total of 11 metaphase plates displaying the mouse-specific number of 40 chromosomes (overall distribution: 26–41 chromosomes). ESC culture techniques have been described previously [58]. Briefly, BK4-G3.16 cells were maintained on mitomycin C-inactivated mouse embryonic fibroblast feeder layers in DMEM supplemented with 15% ESC-tested FBS (PAN Biotech), penicillin/streptomycin, 0.1 mM ß-mercaptoethanol, 2 mM L-glutamine, 0.1 mM non-essential amino acids and 1∶100 LIF produced by recombinant 8/24 720 LIF-D CHO cells [59].

### Transfection and Western blotting

For *EGFP* knockout, U2OS.693 cells in 12-well plates were transfected using the calcium phosphate precipitation method [54] with 75–1200 ng of ZFN expression plasmids, 100 ng of pRK5.mCherry [51] as an internal reference for transfection efficiency, and pUC118 to 1.6 µg. BK4-G3.16 cells were transfected using Lipofectamine 2000 reagent (Invitrogen) according to the manufacturer's protocol. Briefly, the day before transfection 3×10^5^ cells were seeded into a 12-well plate in 1 ml of growth medium without antibiotics. For each sample 1.6 µg of DNA cocktail (as above) was mixed with 5 µl of Lipofectamine 2000. Electroporation of BK4-G3.16 cells was performed as described elsewhere [58]. Briefly, 2–10×10^6^ cells were resuspended in 400 µl DMEM for electroporation (400 V/125 µF; BioRad GenePulser™) with 2–40 µg of ZFN expression plasmids. At the indicated time points, the percentages of EGFP-negative and mCherry-positive cells were determined by flow cytometry (FACSCalibur; BD Biosciences). The graphs always display the absolute fraction of EGFP-negative cells, *i.e.* without background subtraction or normalization for transfection efficiency. For Western blot analysis, HEK-293T cells (ATCC® #CLR-11268™) were transfected using polyethyleneimine (PEI)-mediated transfection as previously described [60] and harvested after 30 h. Thirty µg of lysate was separated via sodium dodecyl sulfate-polyacrylamide gel electrophoresis and transferred to polyvinylidene difluoride membranes. ZFN and EGFP expression was detected simultaneously with antibodies directed against the HA tag (NB600-363, Novus Biologicals) and EGFP (MAB3580, Millipore) and visualized by infrared imaging after incubation with secondary antibodies conjugated with either IR-Dyes 680 or 800CW (LI-COR Biosciences).

### Genotyping

An amplicon encompassing a 556-bp fragment of the *EGFP* gene was produced by PCR using 100 ng of genomic DNA as a template, along with 10 µM of each primer #154 5′-ctacggcaagctgaccctgaa and #598 5′-gaactccagcaggaccatgt), 10 mM dNTPs, and 0.5 U of Phusion High-Fidelity DNA Polymerase (Finnzymes) in 1× reaction buffer for 30 cycles. The generated products were separated by gel electrophoresis, extracted using QIAquick Gel Extraction kit (Qiagen), and subjected to sequencing. The T7 endonuclease I (T7E1) assay was performed as described previously [51]. In brief, 100 ng of the purified 556-bp PCR amplicon (see above) was melted and re-annealed to allow the formation of heteroduplex DNA, treated with 5 U of T7E1 (New England BioLabs) for 20 min at 37°C, and separated on a 2% agarose gel. For TaqI-based genotyping, the 556-bp *EGFP*-specific amplicon was subjected to a nested PCR using primers #850 5′-atcgacttcaaggaggacggc and #597 5′-ggtgctcaggtagtggttgtc. The resulting 228-bp fragment was purified using QIAquick PCR purification kit (Qiagen), followed by digestion with TaqI restriction enzyme (NEB).

### Analysis of metaphase chromosomes and teratoma formation assay

Metaphase spreads were prepared as described previously [61] and an average of 20 spreads was analyzed after Giemsa staining. Teratoma assays were performed as previously described [62].
